# Detection of Vasodilators From Herbal Components by a Transcriptome-Based Functional Gene Module Reference Approach

**DOI:** 10.3389/fphar.2019.01144

**Published:** 2019-10-02

**Authors:** Peng Li, Chang Chen, Wuxia Zhang, Dingrong Yu, Shaoyan Liu, Jinzhong Zhao, An Liu

**Affiliations:** ^1^College of Arts and Sciences, ShanXi Agricultural University, Taigu, China; ^2^Institute of Chinese Materia Medica, China Academy of Chinese Medical Sciences, Beijing, China; ^3^Graduate School of China Academy of Chinese Medical Sciences, Beijing, China

**Keywords:** vasodilator, herbal component, gene expression profile, gene module, drug discovery

## Abstract

Vasodilatation is one of the key therapeutic strategies for the treatment of various cardiovascular diseases with high blood pressure. Therefore, development of drugs assisting blood vessel dilation is promising. It has been proven that many drugs display definite vasorelaxant effects. However, there are very few studies that systemically explore the effective vasodilators. In this work, we build a transcriptome-based functional gene module reference approach for systematic pursuit of agents with vasorelaxant effects. We firstly curate two functional gene modules that are specifically involved in positive and negative regulation of vascular diameter based on the known gene functional interaction knowledge. Secondly, a collection of gene expression profiles following herbal component treatment are collected from a public gene expression database. Then, the correlation of the gene modules is evaluated in each herbal component–induced gene expression profile by gene set enrichment analysis. The vasorelaxant effects of the candidate compounds can be predicted and ordered by the values of a defined index. Finally, the top 10 candidate compounds are experimentally tested for their vasorelaxant effects on vessel contraction induced by Phe in aortic rings. This strategy integrating different types of technologies is expected to help to create new opportunities for the development of novel vasodilators.

## Introduction

Agents that induce or initiate vasodilatation, the widening of blood vessels, are vasodilators, which are frequently used to treat conditions with an abnormally high blood pressure, such as hypertension, angina, and congestive heart failure (Bader and Zolty, 2010; Whelton et al., 2018). Types of vasodilators with different mechanisms, such as calcium channel blocker, angiotensin-converting enzyme (ACE) inhibitors, cGMP-specific 3',5'-cyclic phosphodiesterase (PDE5) inhibitors, and potassium channel openers, have been approved in clinic (Saji et al., 2014). Despite the plethora of vasodilator options, some problems including the universality and severity of high blood pressure, drug resistance, and drug dependence need to be addressed, in part, by development of new drugs (Oparil and Schmieder, 2015). Different from current drugs with specific targets to dilate blood vessels, we aim to build an alternative strategy that is capable of systematically exploring vasodilators by recapitulating the molecular network of vasodilatation.

Systems biology approaches are naturally suited to capture the unbiased, large-scale readouts from complex systems (Berger and Iyengar, 2009). Expression profile–based methods are the most general ones, using transcriptional profiles as signatures of the activity of a given pathway, disease, or compound to discover “connections” between them based on their (anti)correlated transcriptional effects, such as the “Connectivity Map” (Lamb et al., 2006). Inspired by this consideration, it is conceived to build specific functional gene modules of vascular phenotype from the genome-scale functional map. Changes of the modules should be greatly associated with the pathogenesis of vascular diameter. The modules can be used as queries to examine the transcriptional response of drugs to identify candidate vasodilators.

Traditional Chinese medicine (TCM), with natural herbs as major materials, has played a crucial role in human health in China. TCM involves plenty of active ingredients, which are usually used as a source of new chemical entities for modern drug discovery (Tian, 2011). Recent large-scale collaborative efforts have produced compendia of molecular profiles for herbal ingredients (Lv et al., 2017). In the present work, we seek to build a transcriptome-based functional gene module reference (TFGMR) strategy that is able to characterize functional gene modules related to vascular diameter in the transcriptional data of a collection of herbal compounds to evaluate the impact of these compounds on the vascular diameter.

## Materials And Methods

### Gene Expression Data and Preprocess

Gene expression data of herbal compounds were obtained from a systematic study on TCM components by using the gene expression microarray technique (Lv et al., 2017). These small compounds were common in Chinese herbs and TCM formulae, and most of them are the quality control components of TCMs from the *China Pharmacopoeia*. The gene expression data were derived from the human breast cancer epithelial cell line (MCF7) treated with these herbal compounds, profiled using microarray technology with Affymetrix Human Genome U133A 2.0, and collected from the National Center for Biotechnology Information (NCBI) Gene Expression Omnibus (GEO series accession number: GSE85871). The raw data (CEL files) were processed consistently by applying the Affymetrix platform-specific procedure to filter and normalize data sets. Then, for each herbal compound, the differential gene expression values versus control samples were calculated by R package “Limma” (version 3.32.7). The gene list for each compound was produced by ordering genes in a ranked list according to their differential expression values.

### Identification of Functional Gene Module of Regulating Blood Vessel Diameter

Two functional gene modules that positively and negatively regulate blood vessel diameter (PFGM and NFGM) were constructed in this work. Firstly, two Gene Ontology (GO) biological processes (BPs) “positive regulation of blood vessel diameter” (GO: 0097755) and “negative regulation of blood vessel diameter” (GO: 0097756) were collected from QuickGO: a web-based tool for GO searching (http://seek.princeton.edu/) (Zhu et al., 2015). The resulting genes with co-expression score > 0 and *P* value < 0.01 in SEEK were regarded as co-expressed genes of query core genes. Finally, the overlapping genes in two gene sets were removed, and the rest genes composed PFGM and NFGM, respectively (**Supplementary Tables 1** and **2**).

### Enrichment Analysis of Functional Gene Modules in Herbal Compound–Induced Gene Expression Profiles

The enrichments of PFGM and NFGM with herbal compound–induced gene expression profiles were evaluated by using the gene set enrichment approach (GSEA), implemented in the R packages, “GSEA-P” (Subramanian et al., 2005). GSEA calculates the Enrichment Score (ES) for gene sets with respect to the ranked list. The ES value range is [−1 1]. It is a measure based on the Kolmogorov–Smirnov statistics and evaluates the overrepresentation of gene sets at the extremes (top or bottom) of the ranked list. The closer ES is to 1, the closer the genes are to the top of the list (genes tend to be increased in the condition). The closer to −1, the closer the genes are to the bottom of the list (genes tend to be reduced in the condition).

In this work, PFGM and NFGM represent molecular signatures of the blood vessel diameter. To evaluate effects of herbal compounds on the blood vessel diameter, the ES values (ES_PFGM_ and ES_NFGM_) of PFGM and NFGM were measured for each compound-induced gene expression profile. The value TES = ES_PFGM_ − ES_NFGM_ was used as an index to assess the influence of each herbal compound on the blood vessel diameter. For a compound, the larger its TES value, the stronger its ability to dilate blood vessels. The smaller its TES value, the stronger its ability to constrict blood vessel.

### Characterization of the Compound-Regulated Genes in PFGM and NFGM

In this work, for each compound, its regulated genes in PFGM and NFGM are equivalent to the leading-edge subset that is defined to be the core members of the two modules that contribute to the ES in the GSEA method. The leading-edge subset can be interpreted as the core of a gene set that accounts for the enrichment signal. The leading-edge subset of PFGM and NFGM for each compound-induced gene expression profile can be extracted by the method implemented in the R packages, GSEA-P.

### Chemicals and Drugs

Ferulic acid (98% purity), liquiritin (98% purity), magnolol (98% purity), and ginsenoside Rb2 (93.8% purity) were obtained from the National Institutes for Food and Drug Control (NIFDC, Beijing, China). Borneol (98% purity), ginsenoside Rc (98% purity), artemisinin (99% purity), chenodeoxycholic acid (98% purity), daidzin (98% purity), and bacopaside I (98% purity) were purchased from Shanghai yuanye Bio-Technology Co., Ltd. Other reagents were of analytical purity.

### Experimental Animals

Forty male Sprague-Dawley (SD) rats weighing 230–250 g at the age of 7–9 weeks and were purchased from the Animal Breeding Center of Beijing Vital River Laboratories Company (Beijing, China). The rats were maintained under a 12 h light/dark cycle and had free access to food and water. The animals were taken care of by the China Academy of Chinese Medical Sciences’ Laboratory Animal Care Center. All animal experiments were carried out in accordance with the recommendations of institutional guidelines and ethics. The protocol was approved by the Animal Ethical and Welfare Committee, China Academy of Chinese Medical Sciences.

### Preparation of Aortic Rings for Tension Measurements

The *ex vivo* experiment was conducted on thoracic aortas of rats according to the protocol described previously (Chen et al., 2019). Namely, the rats were anesthetized by intraperitoneal injection of chloral hydrate [400 mg/kg body weight (BW)] and were then sacrificed by cervical dislocation. After opening the chest, the rats’ thoracic aortas were obtained and were immediately placed in ice-cold Krebs–Henseleit (KH) solution of the following composition (in mmol/L): NaCl 120, KCL 4.8, MgSO_4_•7H_2_O 1.2, KH_2_PO_4_ 1.2, CaCl_2_ 2.5, NaHCO_3_ 25, and glucose 11 (pH 7.4). The fat tissue adhering to arteries was carefully removed to avoid endothelial cell damage, and the blood vessel was cut into approximately 3-mm-long rings. In the endothelium-denuded experiments, the endothelium was mechanically removed by gently rubbing the luminal surface of the aortic ring back and forth several times with plastic tubing. Aortic rings were suspended in organ baths containing 5 ml KH solution at 37°C gassed with 95% O_2_ + 5% CO_2_, which was maintained constantly throughout the experiments. After equilibration under no tension for 20 min, the aortic rings were allowed to equilibrate for 90 min at a resting tension of 1.0 g. During the equilibration period, the KH solution was changed every 20 min. Changes in tension were recorded by force transducers (FT-102, Chengdu Techman Software Co., Ltd. China) connected to a data acquisition system (BL-420F, Chengdu Techman Software Co., Ltd. China) and were stored in a computer. Moreover, in order to ensure the accuracy and repeatability of the study, 3.0–4.5 g was selected as the inclusion criterion of precontractile force according to preliminary experiments.

### Effects of Herbal Components on Contraction Induced by Phenylephrine

At the steady contraction induced by phenylephrine (Phe) (1 μmol/L) in aortic rings, a cumulative concentration of herbal components (dissolved in KH solution or containing 0.2% DMSO) was added directly to the organ bath, and the effect of the herbal component was recorded. The same volume of KH solution [or containing 0.2% dimethyl sulfoxide (DMSO)] was added to the vehicle control group, and the responses were stopped by washing the aortic rings with fresh KH solution. The effects were expressed as the percentage of relaxation induced by herbal components.

### Data Analysis

Firstly, to assess the significance of the effects of herbal compounds on the vascular phenotype represented by the two functional modules, we computed a nominal *P* value for each TES value by comparing the distribution of the actual value to a null model that obtained TES values for each compound by randomly sampling gene members of PFGM and NFGM 1,000 times with preserved numbers of the two modules (**Supplementary Table 3**). Secondly, the significance of the overlapping analysis between the regulation of the six compounds with relaxant effects was measured by Fisher’s exact test (**Supplementary Table 5**). Finally, the statistical analysis of the functional annotation of gene sets in WebGestalt (http://software.broadinstitute.org/gsea/msigdb/annotate.jsp) follows the instructions of the corresponding website.

Vasorelaxatory activity is expressed as percentage relaxation of Phe (1 µmol/L) precontraction levels. Values are expressed as the means ± SD of the results of the eight samples. Differences across the groups were determined by one-way analysis of variance. Statistical significance was defined as P < 0.05. The concentration for 50% of maximal effect (EC50) values of *in vitro* experiments were obtained by nonlinear regression. Generation of graphs and statistical analysis were performed using GraphPad Prism (GraphPad Software, version 5.01) and SPSS 18.0 software.

## Results

### Transcriptome-Based Functional Gene Module Reference

**Figure 1:**
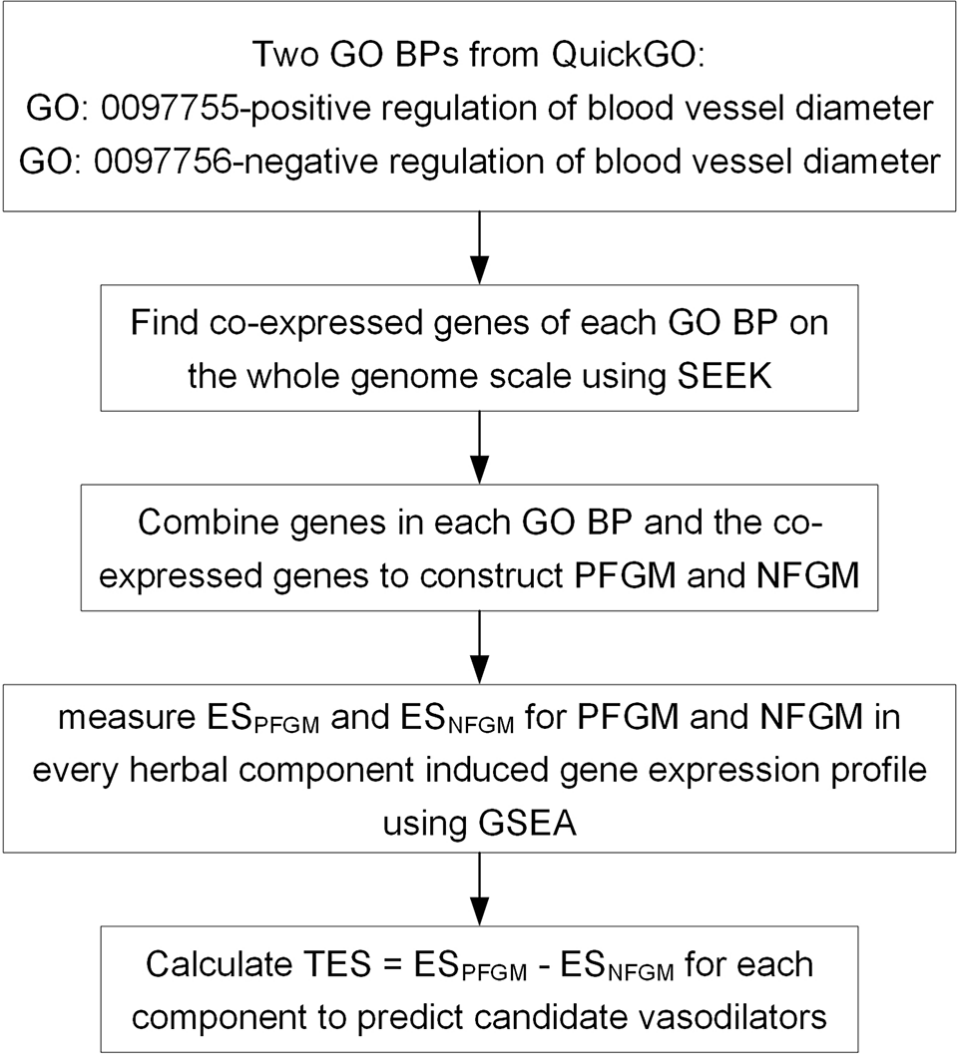
A transcriptome-based functional gene module reference (TFGMR) strategy to predict candidate vasodilators from herbal components based on specific function gene modules of regulating blood vessel diameter and herbal component–induced transcriptional profiles. GO, Gene Ontology; BP, biological process; PFGM, functional gene module that positively regulates blood vessel diameter; NFGM, functional gene module that negatively regulates blood vessel diameter; GSEA, gene set enrichment approach; ES, Enrichment Score.

We present a TFGMR strategy capable of enriching disease-related functional gene modules in drug-induced transcriptional profiles to detect the therapeutic ability of drugs for diseases by regulating specific functional mechanisms. Here, TFGMR is applied to transcriptome resources for 102 herbal components to systematically discover novel vasodilators by regulating blood vessel diameter (**Figure 1**). Firstly, we constructed two functional gene modules PFGM and NFGM that positively and negatively regulate blood vessel diameter. PFGM contains 167 gene members, of which 55 core genes derived from the GO BP “positive regulation of blood vessel diameter” (GO: 0097755) and 112 co-expressed genes of the core genes (**Supplementary Table 1**). NFGM contains 122 members, including 72 core genes from GO BP “negative regulation of blood vessel diameter” (GO: 0097756) and their 50 co-expressions (**Supplementary Table 2**). Then, we examined the enrichment of both PFGM and NFGM to each of the herbal compound–induced gene expression profiles by GSEA approach to assess the effect of each herbal compound on the blood vessel diameter.

### Characterization of Functional Gene Module of Regulating Blood Vessel Diameter

**Figure 2:**
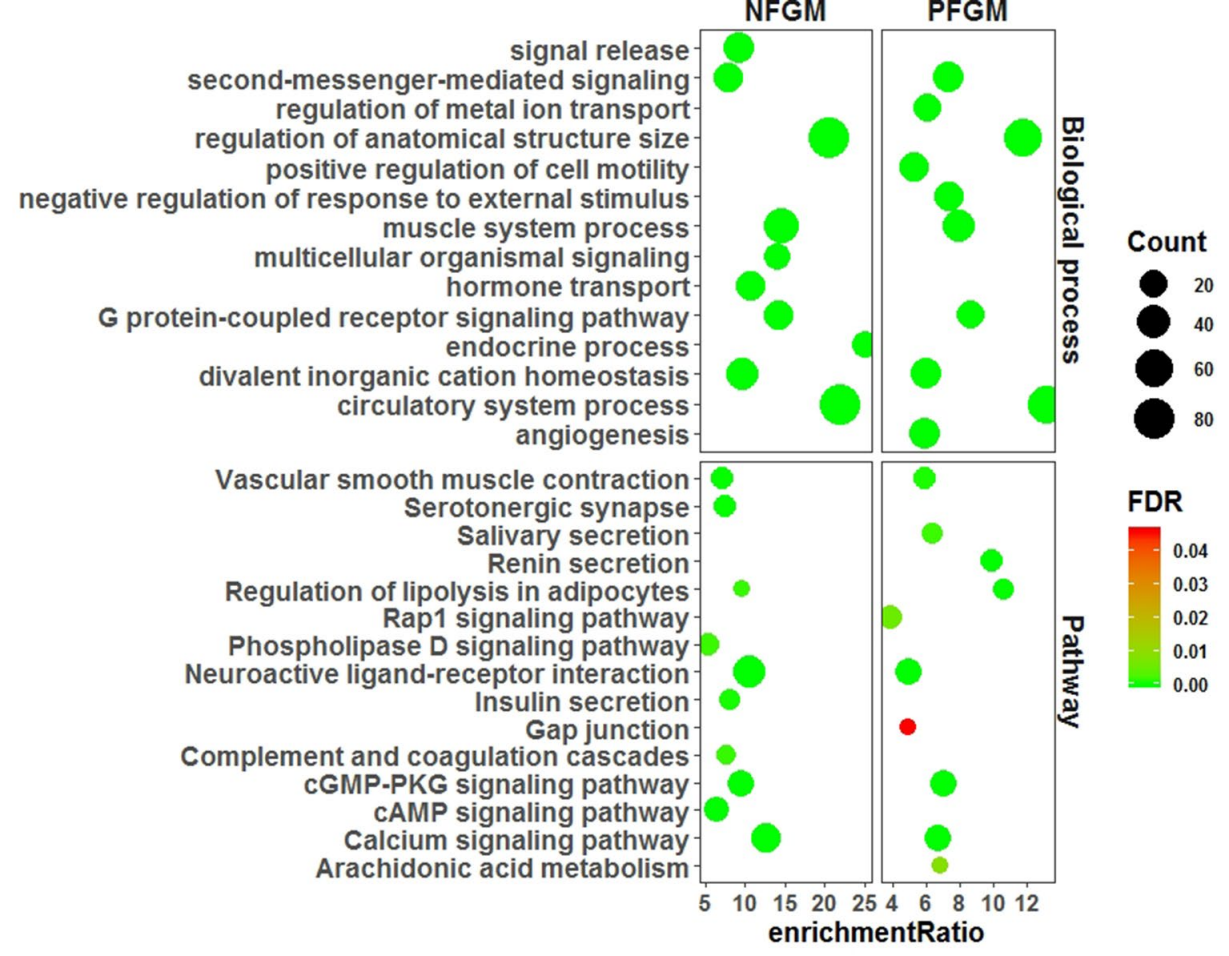
Functional annotations for PFGM and NFGM members.

We characterized PFGM and NFGM with respect to both GO and pathway enrichment to assess the functional association of the two modules with the vascular phenotype by using WebGestalt (Liao et al., 2019). The GO analysis of the top 10 BPs showed that both PFGM and NFGM involve the same BPs of “divalent inorganic cation homeostasis,” “regulation of anatomical structure size,” “circulatory system process,” “muscle system process,” “G protein–coupled receptor signaling pathway,” and “second-messenger-mediated signaling” (**Figure 2**). Similarly, pathway analysis of the top 10 Kyoto encyclopedia of genes and genomes (KEGG) pathways displayed that the two modules were significantly involved in pathways of “regulation of lipolysis in adipocytes,” “cyclic guanosine monophosphate (cGMP)-cGMP-dependent protein kinase (PKG) signaling pathway,” “calcium signaling pathway,” “vascular smooth muscle contraction,” and “neuroactive ligand–receptor interaction” (**Figure 2**). Both GO and pathway terms enriched for PFGM and NFGM are obviously related to the vascular phenotype.

### Predicting Candidate Vasodilators From Herbal Components by TFGMR

Our assumption is that vasodilators should mostly increase the expression of PFGM (ES_PFGM_ > 0) members and decrease that of NFGM (ES_NFGM_ < 0). Therefore, the value TES = ES_PFGM_ − ES_NFGM_ was selected as an index to identify candidate vasodilators. For a compound, the larger its TES value, the higher its capacity to dilate blood vessels. The ordered gene lists were firstly produced for the 102 herbal component–induced gene expression profiles. Then, the GSEA method was utilized to measure the TES value for each herbal compound. The 102 compounds were ordered by their TES values and detailed in **Supplementary Table 3**. The top 10 candidate compounds and their TES values were listed in **Table 1**.

**Table 1:** Top 10 candidate vasodilators predicted by TFGMR and their vasorelaxant effects on Phe-contracted aortic rings.

Herbal component	ES_PFGM_	ES_NFGM_	TES	Concentration (M)	EC_50_ (M)	E_max_ (% Phe)
Ferulic acid	0.19	−0.23	0.42	1.5×10^−4^–4.8×10^−3^	8.9×10^−4^	87.46 ± 2.90
Borneol	0.20	−0.19	0.40	1×10^−8^–1×10^−3^	5.9×10^−6^	87.32 ± 3.55
Liquiritin	0.20	−0.19	0.39	1.1×10^−6^–2.5×10^−4^	2.1×10^−5^	11.10 ± 3.34
Magnolol	0.19	−0.20	0.39	1.1×10^−6^–2.5×10^−4^	1.0×10^−5^	68.94 ± 4.10
Ginsenoside Rc	0.17	−0.21	0.38	5×10^−6^–1.6×10^−4^	2.1×10^−5^	26.74 ± 6.42
Artemisinin	0.17	−0.21	0.38	5×10^−5^–1.6×10^−3^	2.1×10^−4^	52.08 ± 5.23
Chenodeoxycholic acid	0.20	−0.18	0.38	5×10^−5^–1.6×10^−3^	2.0×10^−4^	55.84 ± 4.34
Daidzin	0.19	−0.17	0.36	5×10^−5^–1.6×10^−3^	2.2×10^−4^	84.67 ± 6.56
Bacopaside I	0.17	−0.17	0.34	5×10^−5^–1.6×10^−3^	2.4×10^−4^	15.37 ± 6.25
Ginsenoside Rb2	0.19	−0.15	0.34	2.5×10^−6^–8×10^−5^	9.8×10^−6^	23.27 ± 5.88

TFGMR, transcriptome-based functional gene module reference; PFGM, functional gene module that positively regulates blood vessel diameter; NFGM, functional gene module that negatively regulates blood vessel diameter; ES_PFGM_, enrichment score of PFGM; ES_NFGM_, enrichment score of NFGM; TES, TES = ES_PFGM_- −ES_NFGM_; EC_50_, concentration for 50% of maximal effect.

### The Vasorelaxant Effects of Top 10 Candidate Components on Phe-Contracted Aortic Rings

**Figure 3:**
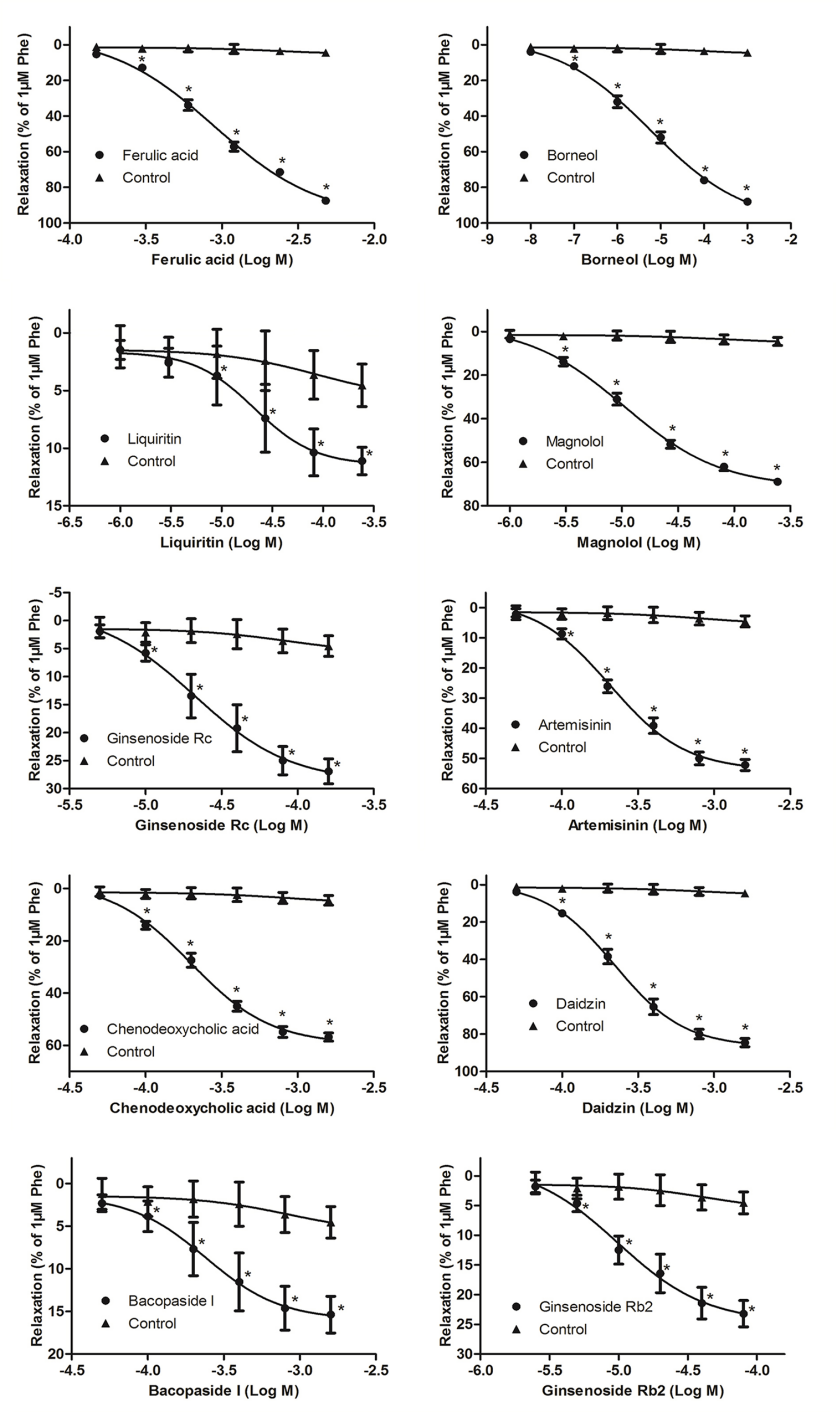
Vasorelaxant effects of various herbal components on rat thoracic aorta rings with endothelium (n = 8) precontracted with Phe. Relaxation (%) was calculated as a percentage of decrease in the maximal tension induced by Phe. Data are shown as the means ± SD. *P < 0.05 vs the control group.

The vasorelaxant effects of the top 10 predicted candidate vasodilators were evaluated on vascular tension of endothelium-intact thoracic aorta rings constricted by Phe. Typical original trace showed that KH solution (control group, contained 0.2% DMSO) did not relax vascular rings preconditioned with Phe (**Supplementary Figure 1**). Compared with the control group, all the 10 herbal components demonstrated significant and dose-dependent vasorelaxant effects on Phe-contracted aortic rings (*P* < 0.05, **Figure 3**). Among them, ferulic acid presented the most powerful vasodilation (E_max_ = 87.46 ± 2.90%), while the minimum potency was elicited by liquiritin (E_max_ = 11.10 ± 3.34%) (**Figure 3** and **Table 1**). This high level on new predictions indicated that the TFGMR strategy is practically useful in prediction of novel vasorelaxant agents and has potential applications in drug development.

### Inferring Mechanisms of Herbal Compounds With Vasorelaxant Effects

**Figure 4:**
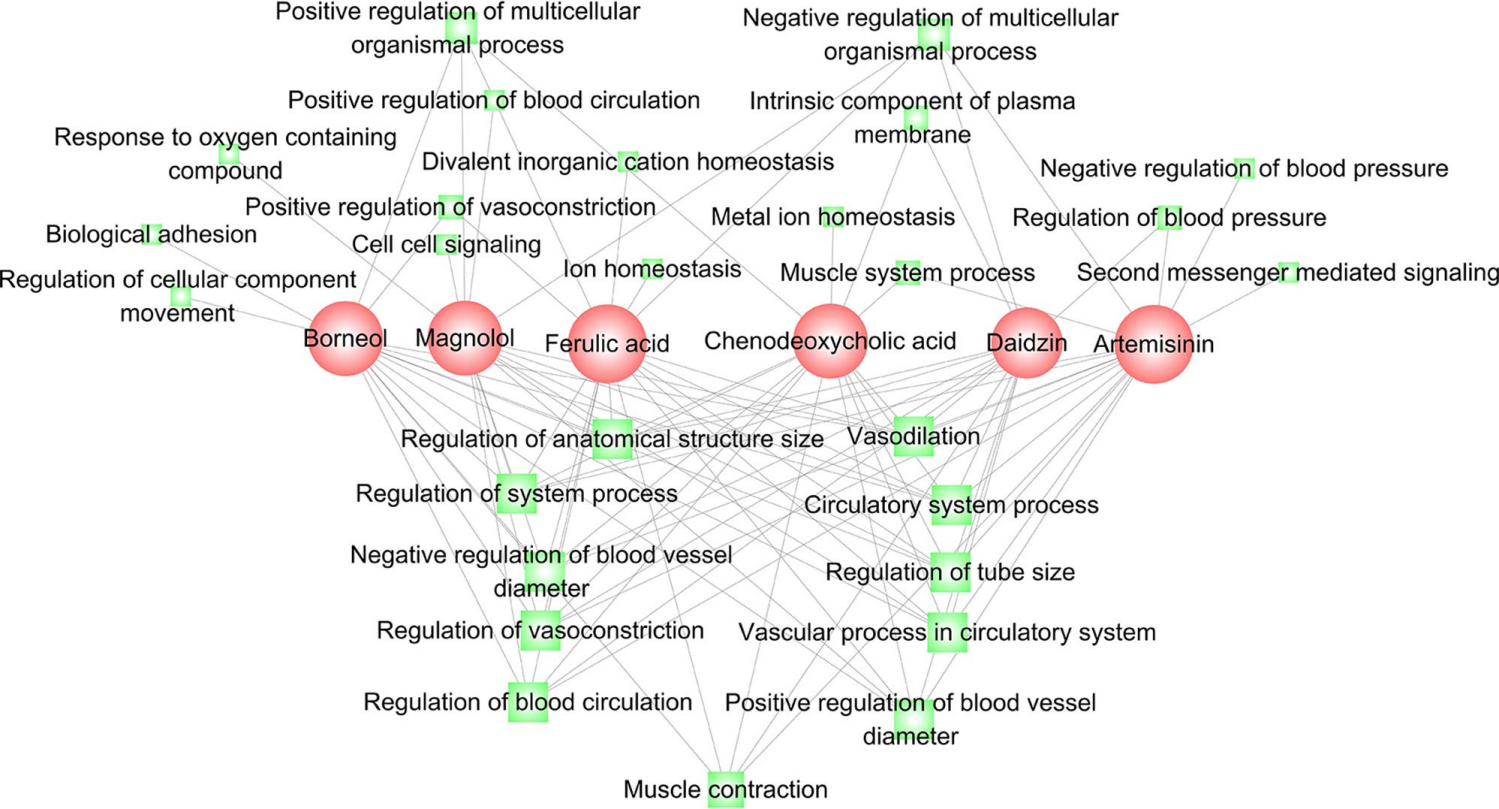
Functional analysis for the genes regulated by the six compounds ferulic acid, borneol, daidzin, magnolol, chenodeoxycholic acid, and artemisinin. A link between a compound (red) and a function node (green) indicates that the regulated genes of the compound are significantly involved in the function annotation. The node size is correlated with the network degree of the node.

The six compounds with obvious relaxant effects [Emax (% Phe) > 50%] were investigated for their corresponding mechanisms based on their regulation on PFGM and NFGM. Firstly, we examined core genes that are significantly regulated in the two modules by the six compounds. For each compound, the regulated genes are the members in the leading-edge subset of the enrichment analysis (see *Materials and Methods*). As shown in **Supplementary Table 4**, ferulic acid, borneol, daidzin, magnolol, chenodeoxycholic acid, and artemisinin influence 67, 59, 50, 58, 59, and 59 genes in PFGM, and 52, 49, 39, 44, 44, and 43 genes in NFGM, respectively. The regulated genes of these compounds were compared, and it was found that the regulated genes for the six compounds significantly overlap with each other (**Supplementary Table 5**, Fisher’s exact test *P* << 0.01), implying that the six compounds share similar mechanisms for their relaxant effects. This was further validated by the functional analysis of these regulated genes of these compounds. In the top 27 enriched GO terms, the regulated genes of the six compounds significantly enriched in eight same GO terms related to vascular phenotype, including vascular process in circulatory system, regulation of vasoconstriction, regulation of tube size, regulation of system process, regulation of blood circulation, regulation of anatomical structure size, and circulatory system process. Besides the similar mechanisms, each compound also has its exclusive mechanism. For example, ferulic acid and chenodeoxycholic acid can regulate genes in GO term “ion homeostasis.” Borneol significantly impact genes in GO terms “regulation of cellular component movement” and “biological adhesion” (**Figure 4** and **Supplementary Table 6**).

## Discussion

High blood pressure contributes to the pathophysiological consequences of most cardiovascular diseases. Thus, inhibiting or reversing high blood pressure is an invaluable strategy for treating cardiovascular diseases and related organ damage (Carey et al., 2018). Although there have been more than 69 drugs in 15 different classes of antihypertensive drugs, such as calcium antagonists, angiotensin-converting enzyme inhibitors, β-adrenoceptor antagonists, diuretics, and direct-acting vasodilators, many more hypertensive patients have resistant hypertension or are uncontrolled for nonadherence or intolerance to available antihypertensive agents (Oparil and Schmieder, 2015). Therefore, novel agents are still needed to fight high blood pressure. TCMs mostly contain hundreds of chemical components, which are the substance basis of TCM pharmacology and provide an abundant source for chemical drug discovery. In this work, we systemically investigated the effects of herbal ingredients on the vascular phenotype by using a transcriptome-based approach, TFGMR.

Vascular tension has been reported to play a critical role in regulating blood pressure. One of the dominating mechanisms of antihypertensive drugs is to reduce the vascular resistance by vasodilation. In the present study, vasorelaxant effects of the top 10 candidate components have been examined. The results showed that all the 10 components have varying degrees of vasodilation. Among them, ferulic acid, borneol, and daidzin had strong relaxant effects [Emax (% Phe) > 80%, **Table 1**], which is in accordance with previous findings (Suzuki et al., 2007; Deng et al., 2012; Silva-Filho et al., 2012; Zhao et al., 2014; Zhou et al., 2017; Santos et al., 2019). Magnolol, artemisinin, and chenodeoxycholic acid elicited a moderate relaxation [Emax (% Phe) > 50%, **Table 1**], also consistent with the reports of the previous studies (Teng et al., 1990; Li et al., 2005; Seok et al., 2012; Wang et al., 2017). In addition, our work first reported the vasorelaxant effects of four herbal compounds, liquiritin, ginsenoside Rc, bacopaside I, and ginsenoside Rb2, although the effect is low (Emax (% Phe) < 30%, **Table 1**). Interestingly, it was found that the vasorelaxant effects of some of these components agreed well with the pharmacological effects of their original herbs (see details in **Supplementary Table 7**). For example, ferulic acid and magnolol are main components of TCM “chuanxiong” (*Ligusticum chuanxiong* Hort) and “houpo” (*Magnolia officinalis* Rehd. et Wils), respectively. In TCM theory, these herbs could promote “Qi” circulation in the body, dispel wind, and eliminate dampness, in line with the theory of vasodilatation (Liu et al., 2017). This finding suggested that the TFGMR strategy is a well-suited model to predict the vasorelaxant effects of TCM.

Gene expression profiles can be used as a signature of the activity of a given drug or disease to find the connections among drugs and diseases using their transcriptional responses (Lamb, 2007; Subramanian et al., 2017). The expression profile–based method has been used for drug repositioning, target prediction, and discovering immunomodulatory effects of drugs (Iorio et al., 2010; Sirota et al., 2011; Kidd et al., 2016; Li et al., 2019). With more fine-grained, large-scale omics data sets becoming publicly available, the present knowledge allows a more accurate delineation of the biological module/network of pathological processes. We here identified and annotated two vessel diameter–related gene modules PFGM and NFGM, by investigating genes involved in the positive and negative regulation of the vessel diameter from a genome-scale gene functional map.

The TFGMR is utilized to characterize the enrichment of PFGM and NFGM in the herbal component–induced gene expression profiles and define a TES value for each herbal component to measure its vasorelaxant effect. The vasorelaxant effects of the top 10 candidate herbal components were examined by *ex vivo* experiments of aortic ring contraction induced by Phe. Among them, six compounds, ferulic acid, borneol, magnolol, artemisinin, chenodeoxycholic acid, and daidzin, elicited an obvious relaxation (E_max_ (% Phe) > 50%, **Table 1**) and may be used as potential therapeutics for diseases with high blood pressure.

It should be noted that although only a database of herbal chemicals was tested by TFGMR, the method is indeed suitable for screening any chemicals for which the actions on the gene expression levels are known. In addition, the candidate compounds should be tested for their direct effects on dilation of resistance vessels, which will be evaluated by laser Doppler flowmetry in further work.

## Data Availability Statement

Publicly available datasets were analyzed in this study. These data can be found here: NCBI Gene Expression Omnibus, GEO series accession number: GSE85871.

## Ethics Statement

The animal study was reviewed and approved by China Academy of Chinese Medical Sciences’ Laboratory Animal Care Center.

## Author Contributions

PL and CC designed research, performed experiments, analyzed data, and wrote the manuscript. WZ, DY, and SL performed experiments and collected and analyzed data. JZ and AL supervised the work and wrote and reviewed the manuscript.

## Funding

This research was supported by the National Natural Science Foundation of China (No. 81703945, No. 31800678) and the Technology and Innovation Fund of Shanxi Agricultural University (No. 2016YJ17, No. 2017YJ40).

## Conflict of Interest Statement

The authors declare that the research was conducted in the absence of any commercial or financial relationships that could be construed as a potential conflict of interest.
