# Supplementary material for: Detection of Vasodilators From Herbal Components by a Transcriptome-Based Functional Gene Module Reference Approach
Source: Front Pharmacol. 2019 Oct 2;10:1144. doi: 10.3389/fphar.2019.01144 (PMC6783510; doi:10.3389/fphar.2019.01144)
Supplement: Supplementary file 8 [file DataSheet_1.pdf]

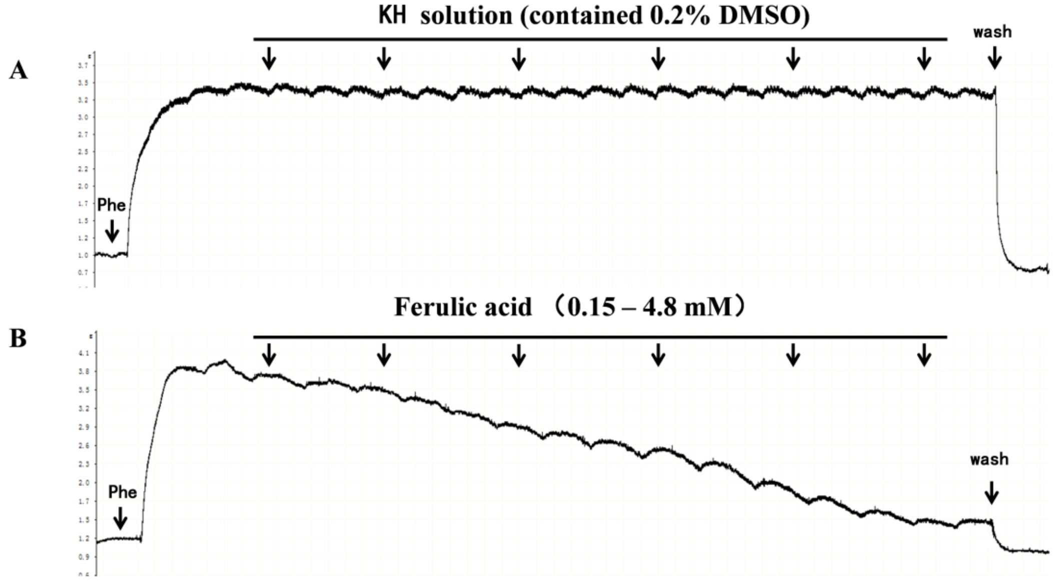

Supplementary figure 1 Representative original recordings of KH solution (contained 0.2% DMSO) or herbal component-induced relaxation (Take ferulic acid for example) on vascular rings precontracted with Phe.
